# Lactic acid bacteria antibacterial and immunomodulatory properties in norm and intravaginal staphylococcosis

**DOI:** 10.1186/1878-5085-5-S1-A137

**Published:** 2014-02-11

**Authors:** Lidia P Babenko, Viktoria V Mokrozub, Olena Yu Sokolvyak, Liudmyla M Lazarenko, Mykola Ya Spivak

**Affiliations:** 1 D.K. Zabolotny Institute of microbiology and virology, National Academy of Sciences of Ukraine, 154, Zabolotny str., Kyiv, D03680, Ukraine

## Introduction

It is known that uncomplicated infections of urinary tract and vaginosis are often caused by opportunistic commensal bacteria of the *Staphylococcus* genus. The newest probiotics based on representatives of the normal commensal microflora – non-pathogenic lactic acid bacteria with antibacterial and immunomodulatory properties may become alternative treatment methods for patients with uncomplicated urinary tract infections and vaginosis.

## Methods

*In vivo* experimental studies were performed on six-week-old female BALB/c mice. Staphylococcosis was modeled through intravaginal administration of the *S. aureus* strain 8325-4 to mice, in doses of 5 x 10^7^ cells per animal. Twenty-four hours after infection, mice were given an intravaginal injection of a probiotic bacteria suspension at a dose of 1 × 10^6^ cells per animal, once per day for 7 days.

## Results

It was found that identified and characterized probiotic strains *L. casei* IMV B-7280, *L. acidophilus* IMV B-7279, *L. delbrueckii* subsp. *bulgaricus* IMB B-728, *B. animalis* VKL and *B. animalis* VKB *in vitro* had different antagonistic effect on museums and clinical strains of pathogenic and opportunistic microorganisms. *L. acidophilus* IMV B-7279, *L. delbrueckii* subsp. *bulgaricus* IMB B-7280 and *B. animalis* VKB showed efficient antagonistic activity. *Staphylococcus* genus bacteria were sensitive to all these probiotic strains. Biocompatibility of lacto- and bifidobacteria was shown, that indicates the possibility of different strain’s compositions creation.

It was shown that intravaginal probiotic strains administration to intact or *Staphylococcus* infected mice resulted in a significant increase in lacto- and bifidobacteria number in the vagina under physiological norm and in cases of staphylococcal infection. This indicates that all of these probiotic strains temporarily colonized mice vagina. Antistaphylococcal activity *in vivo* was confirmed by the significant reduction of *S. aureus* 8325-4 colonies, which were seeded out from infected mice vagina. *L. casei* IMV B-7280 - *B. animalis* VKL - *B. animalis* VKB composition’s antistaphylococcal action was the most effective and more active than in each of strains alone or in different compositions of two or three bacteria.

Immunomodulatory properties of studied strains in monoculture or in different compositions in cases of experimental intravaginal staphylococcosis in mice caused phagocytic cells functional activity increasing, immune cell activation and interferon-γ and IL-12 production normalization in different periods of observation. However, decreased production of IL-4 indicated the ability of these probiotic strains to balance the immune response towards increasing the level of Th1-type cytokines that guide cell type development of the immune response.

## Conclusion

Probiotic strains of *L. casei* IMV B-7280, *L. acidophilus* IMV B-7279, *B. animalis* VKL and *B. animalis* VKB in monoculture or in different compositions are promising to create a highly effective probiotic with antistaphylococcal and immunomodulating activity for farm animals’ treatment from infectious and inflammatory diseases of the urogenital tract, in particular, induced by staphylococci.

